# Supplementary material for: Antioxidant Molecular Brain Changes Parallel Adaptive Cardiovascular Response to Forced Running in Mice
Source: Antioxidants (Basel). 2022 Sep 23;11(10):1891. doi: 10.3390/antiox11101891 (PMC9598430; doi:10.3390/antiox11101891)
Supplement: Supplementary file 1 [file antioxidants-11-01891-s001.zip › antioxidants-1878152-supplementary.pdf]

Supplementary Materials

Antioxidant Molecular Brain Changes Parallel Adaptive Cardiovascular Response to Forced Running in Mice

Clara Bartra, Lars Andre Jager, Anna Alcarraz, Aline Meza-Ramos, Gemma Sangüesa, Rubén Corpas, Eduard Guasch, Montserrat Batlle and Coral Sanfeliu.

Table S1. List of TaqMan® Gene Expression Assays used for real time qPCR analysis

Gene Symbol	Assay ID	Gene Name
Actb	Mm02619580_g1	*Actin, beta*
Bdnf	Mm04230607_s1	*Brain Derived Neurotrophic Factor*
Cat	Mm00437992_m1	*Catalase*
Creb1	Mm00501607_m1	*CAMP Responsive Element Binding Protein 1*
Gdnf	Mm00599849_m1	*Glial Cell Derived Neurotrophic Factor*
Nfe2l2	Mm00477784_m1	*NFE2 Like BZIP Transcription Factor 2*
Psmb5	Mm07296970_g1	*Proteasome 20S Subunit Beta 5*
Psmb6	Mm01245590_g1	*Proteasome 20S Subunit Beta 6*
Psmb7	Mm01327044_m1	*Proteasome 20S Subunit Beta 7*
Psmb8	Mm01278980_g1	*Proteasome 20S Subunit Beta 8*
PsmB9	Mm00479004_m1	*Proteasome 20S Subunit Beta 9*
Psmb10	Mm00479052_g1	*Proteasome 20S Subunit Beta 10*
Sirt1	Mm00490758_m1	*Sirtuin 1*
Sod2	Mm01313000_m1	*Superoxide Dismutase 2*
Ubc	Mm01198158_m1	*Ubiquitin C*
Vegfa	Mm01281449_m1	*Vascular Endothelial Growth Factor*

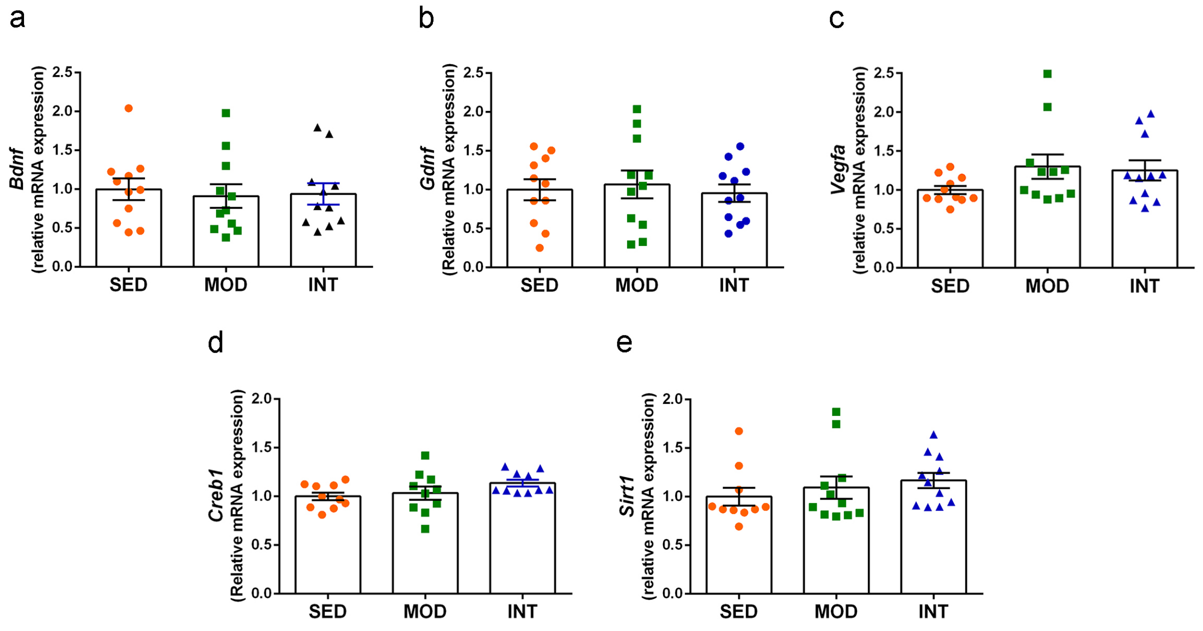

Figure S1. Gene expression of neuroplasticity markers was not significantly increased by exercise training regimens. Genes for neurotrophic factors BDNF (**a**), GDNF (**b**) and VEGF (**c**); and for the signal transducers CREB1 (**d**) and SIRT1 (**e**). Experimental groups: SED (orange circles), sedentary;

MOD (green squares), moderate training; INT (blue triangles), high-intensity training. Values are mean ± SEM ((**a-c**) SED n = 11, MOD n = 11, INT n = 11); (**d**) SED n = 10, MOD n = 10, INT n = 10; (**e**) SED n = 10, MOD n = 11, INT n = 11).

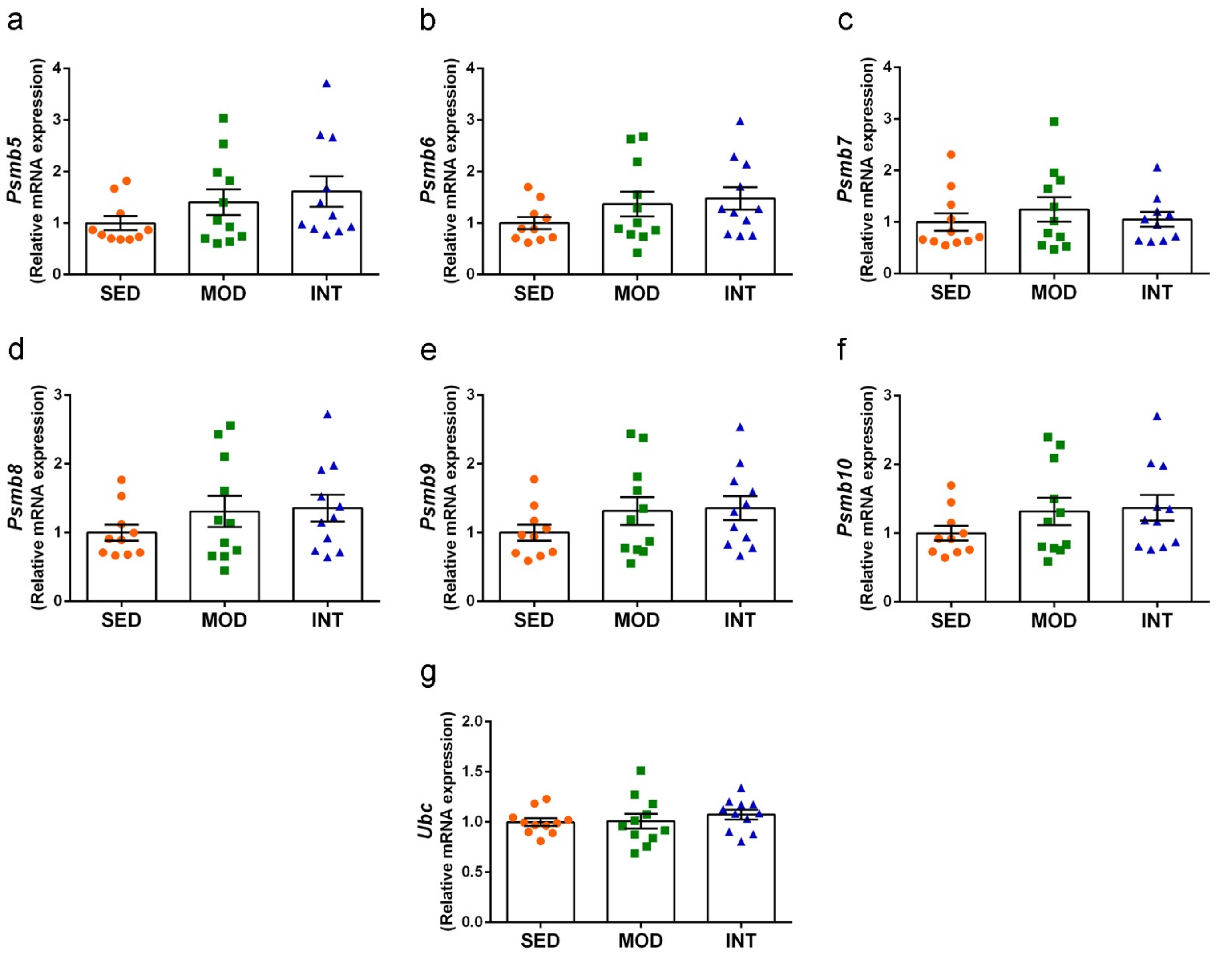

Figure S2. Gene expression of the catalytic 20S constitutive proteasome and 20S immunoproteasome was not significantly increased by exercise training regimens. Genes: *Psmb5* codifies for subunit β5 with chymotrypsin-like activity (**a**); *Psmb6* codifies for subunit β1 with caspase-like activity (**b**); *Psmb7* codifies for subunit β2 with trypsin-like activity (**c**); *Psmb8* codifies for subunit β5i with chymotrypsin-like activity (**d**); *Psmb9* codifies for subunit β1i with caspase-like activity (**e**); *Psmb10* codifies for subunit β2i with trypsin-like activity (**f**); *Ubc* codifies for ubiquitin (**g**). Experimental groups: SED (orange circles), sedentary; MOD (green squares), moderate training; INT (blue triangles), high-intensity training. Values are mean ± SEM ((**a,b,d-f**) SED n = 10, MOD n = 11, INT n = 11); (**c**) SED n = 11, MOD n = 11, INT n = 10; (**g**) SED n = 11, MOD n = 11, INT n = 11).
